# Synaptic ribbons foster active zone stability and illumination-dependent active zone enrichment of RIM2 and Cav1.4 in photoreceptor synapses

**DOI:** 10.1038/s41598-020-62734-0

**Published:** 2020-04-06

**Authors:** Ekta Dembla, Mayur Dembla, Stephan Maxeiner, Frank Schmitz

**Affiliations:** 1 Institute of Anatomy and Cell Biology, Department of Neuroanatomy, Saarland University, Medical School, 66421 Homburg, Germany; 2 Present Address: Institute of Anatomy and Cell Biology, Saarland University, AG Krasteva-Christ, 66421 Homburg, Germany

**Keywords:** Synaptic vesicle exocytosis, Retina

## Abstract

Rod photoreceptor synapses use large, ribbon-type active zones for continuous synaptic transmission during light and dark. Since ribbons are physically connected to the active zones, we asked whether illumination-dependent changes of ribbons influence Cav1.4/RIM2 protein clusters at the active zone and whether these illumination-dependent effects at the active zone require the presence of the synaptic ribbon. We found that synaptic ribbon length and the length of presynaptic Cav1.4/RIM2 clusters are tightly correlated. Dark-adaptation did not change the number of ribbons and active zone puncta. However, mean ribbon length and length of presynaptic Cav1.4/RIM2 clusters increased significantly during dark-adaptation when tonic exocytosis is highest. In the present study, we identified by the analyses of synaptic ribbon-deficient RIBEYE knockout mice that synaptic ribbons are (1) needed to stabilize Cav1.4/RIM2 at rod photoreceptor active zones and (2) are required for the darkness-induced active zone enrichment of Cav1.4/RIM2. These data propose a role of the ribbon in active zone stabilization and suggest a homeostatic function of the ribbon in illumination-dependent active zone remodeling.

## Introduction

The active zone of the presynaptic terminal controls central aspects of presynaptic communication^1,2^. It is a protein-rich, electron-dense specialization of the plasma membrane at which synaptic vesicle fusion preferentially occurs. The active zone is strongly enriched in voltage-gated Cav-channels and active zone proteins, e.g. RIM-proteins, that attach the vesicle fusion machinery in close proximity to the Cav-channels for tight coupling creating rapidly reacting nanodomains^1,3–9^.

Ribbon synapses are continuously active chemical synapses with specialized active zones that are considered to promote both fast, transient and slow, continuous synaptic vesicle exocytosis^10,11^. The active zone of ribbon synapses is extended by large electron-dense structures, synaptic ribbons, that recruit additional vesicles to the active zone. The basal row of ribbon-associated vesicles is positioned close to the Cav-channels and represents the fastest releasable vesicle pool^12^ ideally suited to signal the onset of a stimulus. Synaptic vesicles tethered to the ribbon body, i.e. in some distance from the active zone, are considered to replenish empty release sites at the active zone with slower release kinetics providing information predominantly about the length of the stimulus^12,13^. The main structural component of ribbons is the ribbon-specific protein RIBEYE^14,15^.

Photoreceptor ribbon synapses are the first synapses in the visual system at which light signals are transmitted to the inner retina. Light induces hyperpolarization of photoreceptors and a decrease of exocytosis at the synapse^16^. Rod synapses represent the majority of photoreceptor synapses in the mouse retina and are built in a morphologically fairly uniform manner. They use a single large active zone with a single large ribbon. In EM cross-sections, rod ribbons typically appear as ≈30–50 nm-thick, bar-shaped structures with a height of ≈300–400 nm jutting perpendicularly from the presynaptic membrane into the cytoplasm. At the membrane-anchored end, ribbons obtain a length of up to 2 μm along the active zone^17^. The active zone, as well as the attached ribbon, adopt a horseshoe-shaped appearance due to the invagination of the postsynaptic dendrites into the photoreceptor terminal^18^. The synaptic ribbon is physically associated with the active zone as shown by EM tomography^18^. The active zones at the base of the rod ribbon contain L-type Cav1.4 channels with the pore-forming Cav1.4α1-subunit and auxiliary Cavβ2-/α2δ4 subunits^19–26^. A predominant long RIM isoform in rod photoreceptor synapses is RIM2^27–29^. RIM1, another long RIM isoform, most likely also plays a role as judged by mutations in the RIM1 gene that particularly affect photoreceptors^29–33^ as well as short RIM isoforms (RIM3γ, RIM4γ)^34^. In comparison to rod synapses, cone synapses are larger in size and also possess multiple smaller active zones with smaller, densely clustered ribbons^17,29^.

Since the ribbons and the proper active zones of the photoreceptor synapses are physically linked to each other along the membrane-anchored end of the ribbon^18^, these structures might be interdependent. Therefore, we asked whether alterations in the length of the active zone-associated portion of the ribbon influence Cav1.4/RIM2 protein clusters at the active zone. To address this possibility, we evoked illumination-induced changes of rod synaptic ribbon length and checked for concomitant changes in the size of RIM2/Cav1.4 clusters at the active zone. In order to determine whether the synaptic ribbon itself is directly responsible for the illumination-dependent control of active zone protein clustering, we also analyzed photoreceptor synapses from RIBEYE knockout (KO) mice. In these KO mice, ribbons are absent while generally other presynaptic structures appear unaffected^15^. In contrast to wildtype mice, illumination-dependent changes in the size of the active zone protein clusters were abolished in RIBEYE knockout mice. Our data propose that the synaptic ribbon is involved in the illumination-dependent remodeling of the active zones most likely by controlling the delivery and removal of active zone material.

## Materials and methods

### Animals

Experiments were carried out using C57BL/6J adult mice of either sex (3–8 months of age). Mice were subjected to standard 12 hr light/dark cycle (with light on at 5am) and were provided with water and standard food *ad libitum*. Animal care was in accordance with institutional guidelines and all procedures involving animals were carried out in accordance *with* the relevant guidelines and regulations and have been reviewed and approved by the responsible authority (Landesamt für Verbraucherschutz, Geschäftsbereich 3: Amtstierärztlicher Dienst, 66115 Saarbrücken, Germany). RIBEYE knockout mice used in respective experiments were previously described^15^. Cav1.4 knockout retinal tissue^35^ was kindly provided by Prof. Dr. M. Biel (Munich, Germany) and Prof. Dr. V. Flockerzi (Homburg, Germany). Unless denoted otherwise, mice were picked up from the animal house between 3pm-4pm and sacrificed in the laboratory between 4:30pm and 7pm. Mice were dissected within 5 min post mortem at ≈30 cd/m^2^.

### Light and dark adaptation of mice

For light and dark adaptation experiments, 10–12 weeks old mice were used. To exclude circadian influences, mice were exposed always at the same time (12 am, noon), in parallel, for 4.5 hr either to light (≈30 cd/m^2^) or to a completely dark environment (<0.008 cd/m^2^). The light adaptation experiments thus always started at 12 am (noon) and eyes were removed and processed 4.5 hr later, at 4:30 pm. Mice were anaesthetized with isoflurane and euthanized by cervical dislocation. Isolation of eyes from dark-adapted mice was done in complete darkness under infrared light provided by two infrared illuminators (Conrad Electronics, Model no. CCD-328H; 750965). The standard binocular system used for detection of visible light was replaced by two FJW optical system infrared viewers (Cat. No. 84499A). Two infrared illuminators (Conrad Electronics, Model no. CCD-328H; 750965) were placed close to the dissecting stage from lateral sides together with an additional infrared light (NITECORE, Chameleon series CI6, 850 nm infrared light, 1500 mW). On the microscope stage, the posterior eyecup with the light-sensitive retina was isolated and flash-frozen in liquid nitrogen-cooled isopentane as described^36,37^. For the dark-adapted retinas, also the freezing of the posterior eyecup was performed in complete darkness with the help of infrared light illumination. Further processing of the samples was done as previously described^36,37^. For indicated experiments (analysis of the illumination-dependent kinetics of ribbon- and active zone dynamics), dark-adapted animals were re-exposed to light (≈30 cd/m^2^) for defined periods of time, (i.e. for 20 min, 40 min and 60 min, respectively) before eyes were dissected as described above.

### Generation of photoreceptor-specific RIM1 and RIM2 single knock-out mice from photoreceptor-specific RIM1/2 double knockout mice for the characterization of RIM antibodies

For validation of a RIM2 rabbit polyclonal antibody (RIM2poly) and of a newly generated RIM2-specific mouse monoclonal antibody (4F7), photoreceptor-specific RIM1 or RIM2 single knockout (SKO) mice were generated by breeding floxed RIM1,2 cDKO [RIM1:fl/fl; RIM2:fl/fl^3,27^] carrying the LMOP-Cre transgene^27^ with wildtype C57BL/6J mice to obtain a first generation of heterozygous mice (each with one wild-type and knockout allele for the RIM1 and RIM2 locus, respectively). These heterozygous mice, containing also LMOP-Cre, were back-crossed with floxed RIM1,2 (RIM1:fl/fl; RIM2:fl/fl) mice to generate homozygous conditional knockout alleles for either RIM1 or RIM2 with a heterozygous allele for the respective other RIM gene. The following primers were used for genotyping: RIM1forward: ACGTTTGCAGCAGAGATGC, RIM1reverse: CCTTCCACAGTCTGCATTCC; RIM2forward: GCCAAAGAGTAGAGTGTTGG TGG, RIM2reverse: GGTGTCTGCATCCAGTGGAGC. The presence of the Cre transgene was confirmed with forward primer: GGTTTCCCGCAGAACCTGAA and reverse primer: AGCCTGTTTTGCACGTTCACC.

## Antibodies

### Cav1.4 antibodies

#### Cav1.4-16D9

Mouse monoclonal antibody 16D9 (IgG2b isotype). This antibody was raised against GST-tagged Cav1.4 fusion protein encoding aa1-aa121 of mouse Cav1.4. The PCR insert was amplified from a cDNA encoding mouse Cav1.4 (OMM4760-99847681 [BioCat]) and primers AAAAGAATTCTAATGTCGGAATCTGAAGTC (forward), AAAACCATGGTGTTGGAGTCGTCCTCA (reverse) and cloned into the *Eco*RI/*Nco*I sites of pGEX-KG. Fusion protein expression and purification was performed using standard methods. Immunization of mice, fusion of splenocytes with myeloma cells and subsequent selection of the surviving hybridoma clones was performed at Absea (Beijing, China) using standard methods. The monoclonal antibody 16D9 was used in a 1:50 dilution for immunofluorescence microscopy (final antibody concentration of ≈0.65 mg/ml)

#### Cav1.4 Cterm

This rabbit polyclonal antibody was raised against a GST fusion protein encoding a 259 amino acid long carboxyterminal peptide region of mouse Cav1.4 ranging from H1725 to L1984 of mouse Cav1.4 (NP_062528.2). The respective cDNA was amplified from the above mentioned Cav1.4 clone using forward primer TTTTGGATCCCACAGGAGAAGCTCTGGG and reverse primer TTTTCTCGAGTTAGAGGG CATGGACACAG and cloned into the *Bam*HI/*Xho*I sites of pGEX-KG. The fusion protein was expressed and purified using standard methods and used for immunization. Antibody generation was performed at the central core facility of the Institute for Biochemistry and Molecular Biology of the Saarland University (head: Prof. Dr. Martin Jung). For immunofluorescene (IF) microscopy, the antibody was used in a 1:100 dilution.

### RIM antibodies

#### RIM2 antibodies

RIM2 mouse monoclonal 4F7 (IgG1 isotype). Mouse monoclonal antibody raised against the peptide sequence MEYSWLEQASWHSSEASPMSL (aa544–aa564), that is located directly aminoterminal to the PDZ-domain of RIM2. This antibody is directed against a 21mer peptide that represents a splice site-specific insert (splice site #2) of mouse RIM2α/βa^38^. This splice site insert is specific for RIM2 and present in the long RIM2 variants RIM2α and RIM2β^1,3,39^. Immunization of mice, fusion of splenocytes with myeloma cells and subsequent selection of the surviving hybridoma clones was performed at Absea (Beijing, China) using standard methods. The protein A sepharose-purified mouse monoclonal antibody 4F7 was used in a 1:50 dilution (final antibody concentration of ≈0.72 mg/ml) for IF.

RIM2 rabbit polyclonal (RIM2 poly). The rabbit polyclonal RIM2 antibody was raised against the N-terminal region of RIM2 (aa1-aa325), containing the Zn-finger and the Rab3-binding region. The N-terminal construct of RIM2 was expressed and purified as a MBP-fusion protein. For this purpose, the corresponding cDNA was amplified via PCR with forward primer TTTGGATCCATGTCGGCTCCACTCGG, reverse primer TAAAAG CTTCTATCTCCTTTGTCTTTCATATT and RIM2 cDNA as template and cloned into the *Bam*HI/*Hin*dIII sites of pMal C2 (NEB) using standard methods. The purified fusion protein was used for subcutaneous immunization of rabbits (Pineda Antikörper-Service, Berlin). This antibody is referred to as “RIM2 poly” in the present manuscript. The antibody also detects RIM1^40,41^. For IF, the antibody was used in a 1:500 dilution.

### Further primary antibodies

Anti-Transducin: Gα_t1_ (K-20) (Santa Cruz; sc-389), an affinity purified rabbit polyclonal antibody raised against a peptide mapping within a highly divergent domain of human Gα_t1_ was used in a 1:200 dilution for IF^42,43^.

Anti-RIBEYE(B): Protein A-purified mouse monoclonal antibody against RIBEYE(B) (clone 2D9^44^) was used in a 1:300 dilution (final antibody concentration of ≈ 0.8 mg/ml) for IF.

Anti-RIBEYE(B): Rabbit polyclonal antiserum against RIBEYE(B) domain (U2656^14,45^) was used in a 1:1,000 dilution for IF.

### Secondary antibodies

Secondary antibodies used were chicken anti-mouse IgG conjugated to Alexa488 (Invitrogen, Carlsbad, CA, USA, #A21200), donkey anti-mouse IgG conjugated to Alexa568 (Invitrogen, #A10037), chicken anti-rabbit IgG conjugated to Alexa488 (Invitrogen, #A21441), and donkey anti-rabbit IgG conjugated to Alexa568 (Invitrogen, #A10042).

### Affinity purification of antibodies using protein A sepharose beads

Antibodies were purified with protein A-sepharose beads. In brief, 50 μl sepharose beads (Sigma, P3391-1G) were washed three times with PBS. Then, 50 μl antibody (i.e. antiserum or ammonium sulphate-precipitated monoclonal antibody (precipitated as previously described^46^) was added to the protein A-sepharose beads that were suspended in 200 μl PBS. The mixture was incubated overnight at 4 °C under mild agitation. On the next morning, protein A sepharose beads with the bound antibody were sedimented by gravity (on ice for 20 min). Beads were washed two times with cold PBS at 13,000 rpm (1 min, 4 °C). After the last spin, the PBS was carefully removed. Next, 200 μl 0.2 M glycine (pH 2.7) was added to the beads and incubated at 4 °C for 5 min for antibody elution. Beads were centrifuged at 13,000 for 1 min at 4°C and the supernatant was collected in a new reaction tube. The eluted antibody was neutralized by the addition of 50 μl 1 M Tris-HCl, pH 8.5. The concentration of purified antibodies was quantified using Bradford reagent. BSA was added for stabilization to a final concentration of 0.5 mg/ml. Affinity-purified antibody was stored at 4°C until further use.

### Immunolabeling of resin sections

For confocal imaging, immunolabeling was performed using 0.5 μm thin (“semi-thin”) resin sections that were placed on glass coverslips, as previously described^36,37,44,47^. For Super-Resolution Structured Illumination Microscopy (SR-SIM) imaging, 1.5 μm thin resin sections were used to cover the entire synaptic terminal of a rod synapse. Processing of the resin sections was carried out similarly as previously described^36,37,44^. To remove the resin, resin sections were incubated with sodium methanolate (30% solution in methanol; Merck) for 12 min; 1:1 mixture of xylol/methanol for 12 min; acetone (2x) for 12 min; water for 2–3 min and PBS for 5 min. Afterwards, sections were incubated with respective primary antibodies at the indicated dilutions (at 4°C overnight). After several washes with PBS, binding of the primary antibodies was detected by incubation with corresponding secondary antibodies conjugated to the indicated fluorophores (1:1,000 dilutions; 3 hr, room temperature). Immunolabelled sections were embedded in antifade, as described before^36,37,44,47^. Negative controls were done by omitting primary antibodies and by using incubations of knockout sections, as indicated.

### Confocal microscopy and quantitative analyses of fluorescence intensity and counting of immunolabelled puncta

Confocal microscopy was performed largely as previously described^37,44^. Confocal images were acquired with an A1R confocal microscope (Nikon) equipped with the NIS Elements software (NIS Elements AR 3.2, 64 bit). A 60X/1.40 N.A. oil objective was used for image acquisition with the 488 nm and 561 nm laser excitation lines. For quantitative analyses of experimental and control retinas, confocal images were acquired under identical conditions by using the ‘‘re-use’’ settings option of the NIS elements software, as previously described^37,44,47^. Quantitative analyses were performed in a blinded manner with the experimenter not knowing the identity of the samples. Fluorescence intensity was measured as integrated density using Fiji (ImageJ 1.5n, NIH). To analyze the fluorescence integrated density, a rectangular region of interest (ROI) was selected directly adjacent to the RIBEYE immunosignals in the OPL using the ROI manager because RIBEYE immunosignals indicate the photoreceptor synapses in the OPL^14^ and, thus, were used as a reference to define the synapse layers. Identical ROIs (ROIs identical in size; ROI copy/pasted from one image to the other for the quantitative measurements) were used for light- and dark-adapted retinal sections. Integrated densities were also determined for these areas. Puncta were counted on the same images that were also analyzed for measuring integrated density. To automatically count the immunolabelled puncta, the plugin “3D object count” from Fiji was used. For the quantification of the confocal microscopy results (performed as described above), we did not discriminate between rod and cone photoreceptor synapses because both synapses are immunolabelled by RIBEYE antibodies and are contained in the OPL (though in different sublayers). Since cone synapses represent less than 5% of total photoreceptor synapses in the murine retina^29,48,49^, the quantification results obtained by confocal analyses can be mainly attributed to rod synapses, the predominant synapse population in the OPL.

## Super-Resolution Structured-Illumination-Microscopy (SR-SIM)

Super-Resolution Structured-Illumination-Microscopy (SR-SIM) was performed as previously described^36,37,44^. For SR-SIM, the ELYRA PS1 setup was used from Carl Zeiss Microscopy GmbH. Images were acquired with a 63X/1.4 NA oil (DIC) objective using 488 nm and 561 nm laser lines and collected through an Andor iXon EM-CCD camera (100 ms exposure time). Both rod and cone synapses are localized in the outer plexiform layer (OPL). The rod synapses constitute the largest portion of photoreceptors in the OPL with the cone synapses representing less than 5% of the number of photoreceptor synapses^29,48,49^. For the SR-SIM analyses of single ribbons, we focused on the synaptic ribbons of rod synapses. Rod synapses possess a single synaptic ribbon that is characterized by its large, horseshoe-shaped appearance^29^. Cones possess multiple, densely clustered synaptic ribbons that are smaller in size than rod synaptic ribbons^29^. Rod synapses are located closer to the outer nuclear layer (ONL). Cone terminals are much larger than rod terminals (typically 3–5 μm in diameter in comparison to 1.5 μm of rod terminals) and are located closer to the inner nuclear layer (INL) than rod synapses. These criteria allow fairly safe discrimination of ribbons from rod and cone synapses. In the SR-SIM analyses, we analyzed the single large ribbons in the OPL which thus predominantly reflect synaptic ribbons from rod synapses. Since we did not use additional cone synapse markers, an exceptional contribution of cone ribbons to the SR-SIM quantification results cannot be completely excluded. But the contribution of cone synaptic ribbons to the SR-SIM quantification results should be very small based on the described reasons.

### Length and area measurement of immunolabelled synaptic ribbons and active zone proteins by 3D SR-SIM

For the measurement of RIBEYE, RIM2 and Cav1.4 puncta lengths, z-stacks (interval of 0.2 μm) were acquired from 1.5 μm thin sections with super-resolution structured-illumination-microscope by using an Elyra setup, as described previously^37,44^. 3-dimensional (3D) images were constructed from the processed data and axial informations were extracted from the 3D images of individual synaptic terminals both for RIBEYE and Cav1.4 using the Zen2012 black software (version 8.0). The maximum 2D image projections were obtained from the 3D images. For contour length measurement, the open poly-line tool was used to determine the length of the RIBEYE, RIM2 and Cav1.4 immunosignal puncta. For area measurements of the maximum 2D projections of the 3D images, the closed Bezier tool was used to mark the boundary along the periphery of the indicated immunosignals to determine the area of the respective structures. Prior to analysis, all the images were subjected to background subtraction using Fiji (ImageJ 1.5n). Background subtraction was manually determined by making an ROI at one corner of the image (without immunosignal) and then subtracting this value from the immunosignals. Quantitative analyses were performed in a blinded manner with the experimenter not knowing the identity of the samples.

### Statistical analysis

Statistical analysis was done using OriginPro 2018 software (OriginLab Corporation). Initially data were subjected to the normality check using the Shapiro-Wilk test. Two-sample T test was used when data were normally distributed. When data were not normally distributed, the Mann-Whitney and Kolmogorov-Smirnov tests were applied. For dark-light stimulus experiments, one-way ANOVA test (at a significance level of 0.05) with Bonholm (Bonferroni-Holm) adjustments for multiple comparisons were applied to compare different groups using OriginPro 2019. Before testing for significance of differences between different experimental groups (e.g. light- versus dark-adapted retinas), it was verified that the results from the individual experiments within the same group (e.g. light-adapted retinas) did not differ significantly from each other. After this verification, data from the individual experiments within the same group were pooled before statistical comparison with the indicated reference group. The same procedure was also used for the statistical analysis of RIBEYE/Cav1.4 length ratios. Differences were considered to be statistically significant with p < 0.05. For all the experiments, the identity of samples was not known to the experimenter.

## Results

### Antibody validation

In the present study, we used antibodies against RIBEYE to label the synaptic ribbons and antibodies against voltage-gated Cav-channels (Cav1.4) and RIM2 to label the active zone of rod photoreceptor synapses.

**Figure 1:**
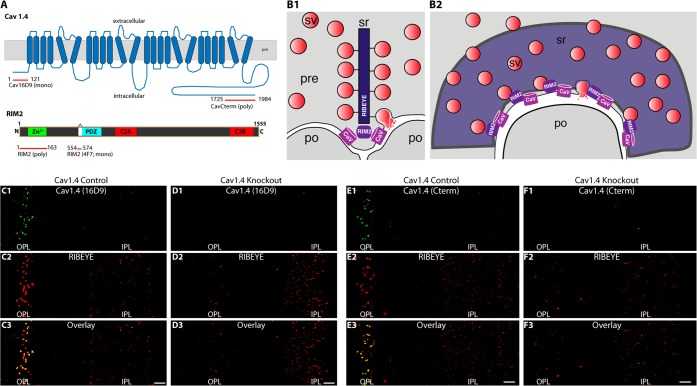
(**A**) Schematic domain structure of Cav1.4 and RIM2. The peptide regions are depicted against which the different antibodies were generated. (**B1**,**B2**) Presumed synaptic relationship between synaptic ribbon, RIM2 and Cav1.4 in a cross-sectioned rod photoreceptor synapse containing a single large synaptic ribbon at a single active zone (sr) (**B1**). (**B2**) corresponds to (**B1**) but visualized in a lateral view. The lateral view reveals the large horseshoe-shaped active zone along the horseshoe-shaped synaptic ribbon. (**C**–**F**) Characterization of two different Cav1.4 antibodies (anti-Cav1.4 16D9 (**C**,**D**); anti-Cav1.4 Cterm (**E**,**F**)). The antibodies were tested on control retina (**C**,**E**) and Cav1.4 knockout retina (**D**,**F**). Semi-thin resin sections were immunolabelled with the indicated antibodies and analyzed by confocal microscopy. No background subtraction was applied to the images. Abbreviations: sr, synaptic ribbon; sv, synaptic vesicle; pre, presynaptic; po, postsynaptic; pm, plasma membrane; OPL, outer plexiform layer; IPL, inner plexiform layer. Scale bars: 5 μm.

For immunolocalization of Cav1.4, we used mono- and polyclonal antibodies raised against the indicated different protein regions of Cav1.4 (Fig. 1A). Cav1.4 is enriched at the photoreceptor synapse active zone at the base of the ribbon^17,19,27–29^. The localization of Cav1.4 and RIM2 is schematically summarized in Fig. 1B1,B2. The specificity of the different Cav1.4 antibodies was analyzed by immunolabelling of wildtype control and Cav1.4 knockout retinas^35,50^ (Fig. 1C–F). In control retinas, we observed strong immunosignals in the outer plexiform layer (OPL), where the photoreceptor synapses are located (Fig. 1C,E). The Cav1.4 immunosignals in the OPL were completely absent in the retinas of Cav1.4 knockout mice (Fig. 1D,F). This was the case for the two different Cav1.4 antibodies used in the study (Fig. 1C–F) demonstrating their specificity for Cav1.4. Sections were co-immunolabelled with antibodies against RIBEYE (Fig. 1C–F) as reference^14^. In the Cav1.4 knockout retina, RIBEYE immunosignals were still present in the IPL and -in an altered distribution and abundance- also in the OPL (Fig. 1D,F). These latter findings are in agreement with previous studies that observed reduced numbers and abnormally looking/distributed ribbons in photoreceptors of Cav1.4 knockout mice^21,35,50–58^. Data from these previous studies were obtained either from the Cav1.4 knockout mouse model^35,50^ that was also analyzed in our study, as well as from other genetically engineered or spontaneous Cav1.4 mouse mutants^51,52,59,60^. We did not observe Cav1.4 immunoreactivity in the inner plexiform layer (IPL) of the retina with both of our Cav1.4 knockout-verified Cav1.4 antibodies. This result was also obtained in other recent studies that applied Cav1.4 antibodies which were verified on Cav1.4 knockout tissue for their specificity to Cav1.4^24,35,56,61^.

Some initial studies found Cav1.4 immunoreactivity also in the IPL of the retina^51,52,62–64^. In addition to a strong Cav1.4 staining of photoreceptor synapses in the OPL, some weaker, diffuse Cav1.4 staining was observed in the IPL of 12–18 μm-thick cryostat sections. These cryostat sections were obtained from retinal tissue that was previously fixed with 4% paraformaldehyde (PFA). In our study, we used much thinner retina sections (0.5 μm-thin sections) for achieving improved resolution and, on purpose, these sections were obtained from chemically unfixed retinas to minimize PFA-induced background staining. Using this procedure, we did not observe any Cav1.4 staining of the IPL with our knockout-verified Cav1.4 antibodies, in line with other recent studies^24,61^. These findings suggest that the expression of Cav1.4 in synapses of the IPL is much lower than in photoreceptor synapses of the OPL and that Cav1.4 expression in the retina is essentially restricted to the OPL. This conclusion has been recently drawn also by other groups^26,50^.

**Figure 2:**
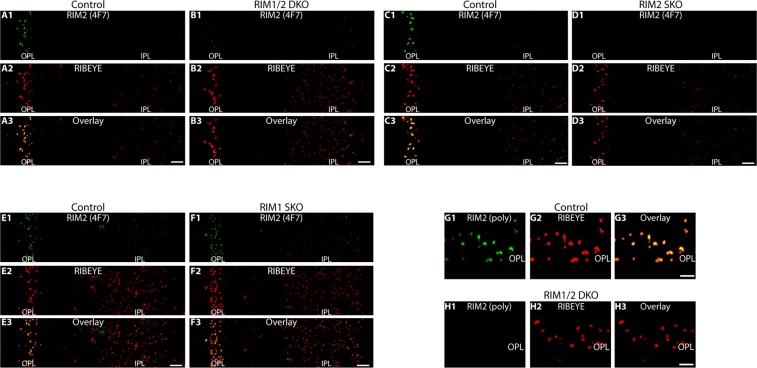
Characterization of two RIM antibodies (anti-RIM2 (4F7); anti-RIM2 (poly)) that were generated against sequence stretches of RIM2 summarized in Fig. 1A. The antibodies were tested on control retina (**A**,**C**,**E**,**G**), RIM1 single knockout (KO) retina (**F**), RIM2 single knockout (KO) retina (**D**) and RIM1/2 double knockout (KO) retina (**B**,**H**). Semi-thin resin sections were double-immunolabelled with the indicated antibodies and analyzed by confocal microscopy. Abbreviations: KO, knockout; SKO, single knockout; DKO, double knockout; OPL, outer plexiform layer; IPL, inner plexiform layer. Scale bars: 5 μm (**A**–**F**); 2 μm (**G**,**H**).

RIM2, a major long RIM isoform in photoreceptor synapses^27,28^ is localized at the base of the synaptic ribbon in photoreceptor synapses^19,28^ [location graphically summarized in Fig. 1B1,B2]. The specificity of the mono- and polyclonal RIM2 antibodies used in the present study were similarly assessed as described above using 1.) photoreceptor-specific RIM1/2 double knockout mice, 2.) photoreceptor-specific RIM1 single knockout mice or 3.) photoreceptor-specific RIM2 single knockout mice (Fig. 2). Single knockouts were used to further test for cross-reactivity of the RIM2 antibodies with RIM1. In this study, we generated a novel RIM2 (4F7) mouse monoclonal antibody that is specific for RIM2. This antibody led to a strong synaptic immunosignal in the OPL of control mice (Fig. 2A,C,E) and photoreceptor-specific RIM1 single knockout mice (Fig. 2F) that was completely absent in the OPL of photoreceptor-specific RIM2 knockout mice (Fig. 2D) and photoreceptor-specific RIM1/2 double knockout mice (Fig. 2B). Despite the absence of RIM2 immunosignals in the OPL of RIM1/2 double knockout retina and in the RIM2 single knockout retina (Fig. 2B1,D1) the retinas produced a robust staining with anti-RIBEYE. The 4F7 monoclonal antibody does not cross-react with RIM1 because retinas of RIM1 single knockout mice immunolabelled with 4F7 (Fig. 2F1) appeared virtually identical to those of control retinas (Fig. 2E1). Synapses in the IPL were immunolabelled by the RIM2 antibody 4F7 both in control mice (Fig. 2A,C,E) as well as in the described knockout mice (Fig. 2B,D,F), as expected, because deletion of the respective genes is specific for photoreceptors and should not affect the inner retina. The rabbit polyclonal RIM2 antibody produced strong immunosignals in the OPL of control retinas (Fig. 2G) that was completely absent in photoreceptor synapses of RIM1/2 double knockout retinas (Fig. 2H). Synapses were co-labelled with anti-RIBEYE as a reference (Fig. 2G,H). RIBEYE immunostaining was qualitatively similar in RIM1/2 double knockout retinas and control retinas (Fig. 2G,H).

### Active zone and ribbons are co-aligned structures in wildtype control mice

**Figure 3:**
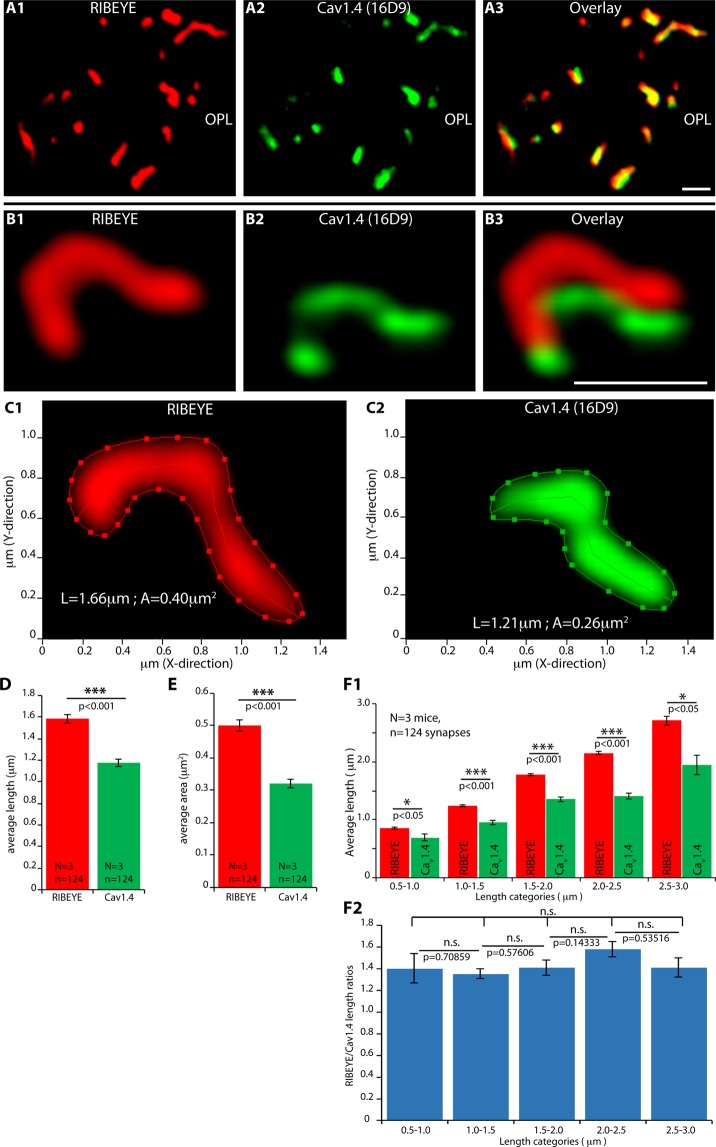
Confocal (**A**) and SR-SIM (**B**,**C**) analyses of rod photoreceptor synapses in the OPL immunolabelled with rabbit polyclonal antibodies against RIBEYE and mouse monoclonal antibodies (16D9) against Cav1.4. From the immunolabelled ribbon/active zone complexes, quantitative parameters (length [central line of the ribbon in C, shown as an example] and area [outer line, surrounding the immunosignal in **C**, shown as an example]) were determined (**D**,**E**). In (**F1**), all synaptic ribbons were subdivided into distinct length classes and analyzed for the respective length of the associated active zone (as measured by the length of Cav1.4-immunosignals). In (**F2**), the ratio between ribbon length/Cav1.4 length is displayed. Values are plotted as mean ± S.E.M. In (**D**,**E**,**F1**,**F2**). Abbreviations: OPL, outer plexiform layer. Scale bars: 1 μm.

Next, we tested whether photoreceptor synaptic ribbons and proper active zones could be distinguished from each other at the light microscopic level using super-resolution structured-illumination microscopy (SR-SIM) of immunolabelled retina resin sections. For this purpose, ribbons were immunolabelled with anti-RIBEYE and active zones of rod synapses with anti-Cav1.4 (Fig. 3). With confocal microscopy (Fig. 3A), the immunosignals of ribbons and active zones (Fig. 3A1,A2) largely overlapped (Fig. 3A3), as expected, due to the limits of resolution of conventional light microscopy. With SR-SIM, the immunosignals of ribbons and Cav1.4 could be clearly separated (Fig. 3B). Rod ribbons could be easily identified by SR-SIM due to their typical shape and size and clearly distinguished from cone ribbons. Rod synapses possess only a single active zone with a single large ribbon. Cone synapses are much less numerous (less than 5% in the mouse retina), positioned closer to the inner nuclear layer and possess multiple, much smaller ribbons. Only rod synapses were included in the SR-SIM analyses. In the rod synapses, RIBEYE and Cav1.4 immunosignals were perfectly co-aligned to each other in parallel stripes (Fig. 3B,C). The RIBEYE/Cav1.4 immunosignals did not completely overlap although they were located close to each other, as expected. The mean ribbon length that we measured by SR-SIM (mean: 1.59 ± 0.04 μm) was remarkably similar to values of ribbon length determined by FIB-SEM^65^. Quantitative analyses revealed that the size of the active zone, as judged by Cav1.4 clusters, was slightly smaller than the contour length and total area of the attached synaptic ribbon (mean ribbon length: 1.59 ± 0.04 μm; mean Cav1.4 length: 1.18 ± 0.03 μm; mean ribbon area: 0.5 ± 0.02 μm^2^; mean Cav1.4 area: 0.32 ± 0.01 μm^2^; Fig. 3D,E). In the 1.5 μm thin section, shorter and longer ribbons were present (Fig. 3F). Therefore, the total population of ribbons was sub-divided into classes of increasing ribbon length and the corresponding active zones length of these ribbons were determined separately (Fig. 3F1). Remarkably, in all these ribbon length classes, irrespectively whether shorter or longer ribbons were analyzed, the ratio between the synaptic ribbon length and the size of the active zone remained largely constant (Fig. 3F2). The different sizes of ribbons appeared to be linked to the size of the corresponding Cav1.4 immunosignals arguing for a tight coupling between ribbon length and active zone length.

### Active zone clusters of RIM2 and Cav1.4 are larger in photoreceptor synapses of dark-adapted retinas in comparison to light-adapted retinas

To further analyze the possibility that ribbon length and active zone length (as judged by RIM2/Cav1.4 cluster size) are coupled, we analyzed rod photoreceptor synapses from light- and dark-adapted mice. Based on previous electron microscopic data, we expected illumination-dependent changes in ribbon size^66–68^; but see^69^. Dark-adaptation of the mice was performed in complete darkness (for 4.5 hrs) and the dark-adaptation was starting always at the same time (12 am, noon). In parallel to the dark-adapted experimental group, a control group of littermate animals was left at ambient illumination for the same time period and sacrificed at the same time as the dark-adapted mice to avoid any influence of a circadian rhythm. Although mice were kept and dissected in complete darkness, dark-adapation of the retinas was further verified by analyzing the distribution of α-transducin (data not shown). α-transducin can be used as a marker for the state of light-/dark-adaptation because it migrates from the outer segments into the photoreceptor inner segments in a light-dependent manner^70,71^. In the light-adapted samples analyzed in the study, α-transducin was always translocated into the inner segments in comparison to the corresponding dark-adapted samples.

**Figure 4:**
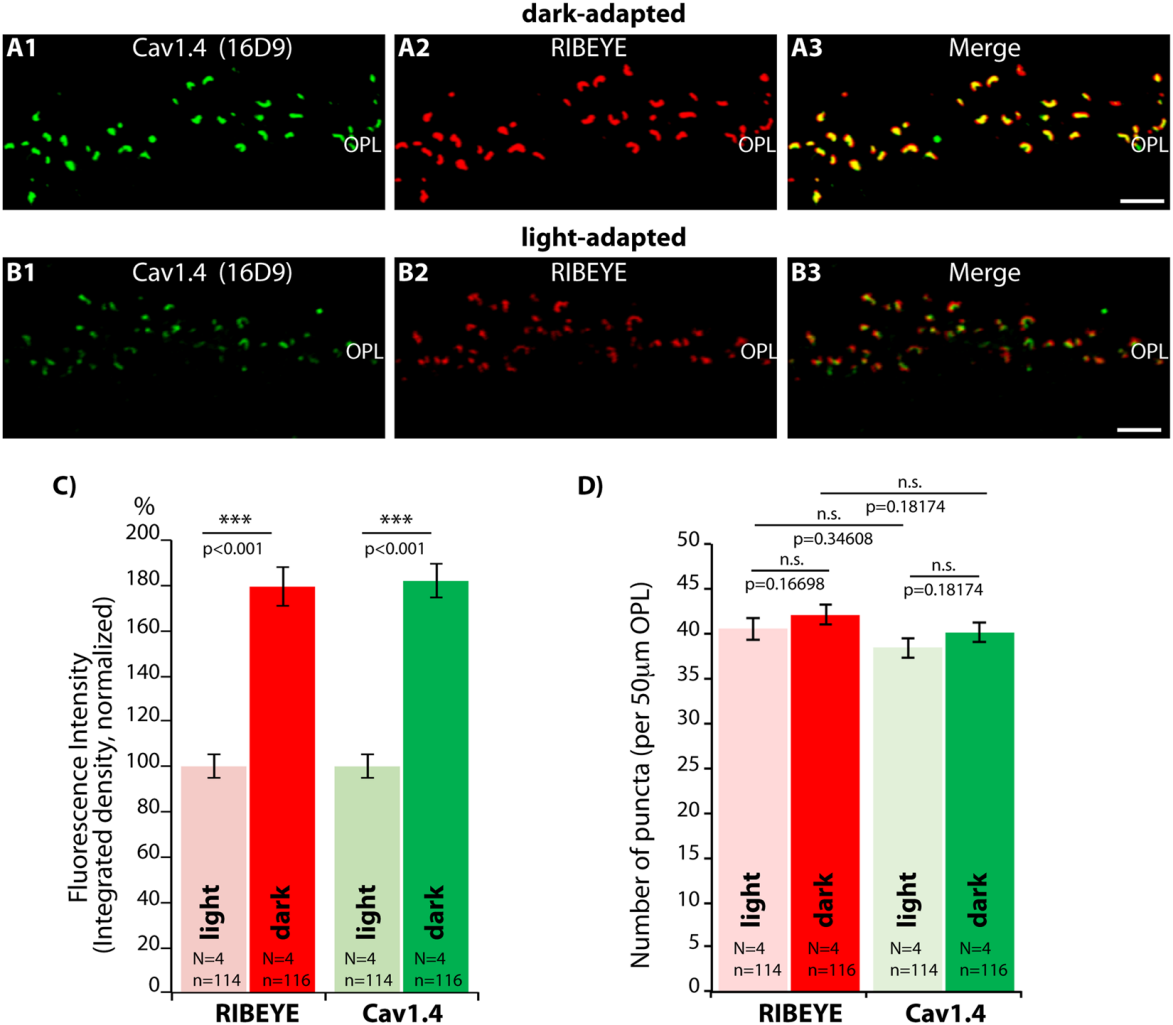
Confocal analyses of photoreceptor synapses from light- and dark-adapted retinas immunolabelled with polyclonal antibodies against RIBEYE (U2656^14^) and mouse monoclonal antibodies against Cav1.4 (16D9). (**A**,**B**) Representative confocal images of dark- and light-adapted retinas; (**C**,**D**) quantitative analyses of the strength of the immunofluorescence signals (measured as integrated density) in (**C**), quantitative analyses of immunolabelled puncta in (**D**). Values are plotted as mean ± S.E.M. In (**C**,**D**). Abbreviations: S.E.M., standard error of the mean; OPL, outer plexiform layer. Scale bars: 5 μm.

From confocal analyses of the respective immunolabelled sections (representative images shown in Fig. 4A1–3,B1–3) we quantitatively analyzed the fluorescence intensity of the RIBEYE and Cav1.4 immunosignals in the OPL (as integrated density; Fig. 4C). Immunosignals for both RIBEYE and Cav1.4 were stronger in the OPL of dark-adapted retinas in comparison to light-adapted retinas (Fig. 4C) while the number of immunoreactive RIBEYE/Cav1.4 puncta remained the same (Fig. 4D). These findings demonstrate that the number of active zones and synaptic ribbons are identical in dark- and light-adapted retinas. Cone synapses represent only a minority of synapses in the mouse retina with less than 5% of the total number of photoreceptor synapses in mice^29,48,49^. Therefore, the observed strong differences in immunosignal intensities are most likely caused by changes in rod synapses, the predominant synapse population in the OPL of the mouse retina.

**Figure 5:**
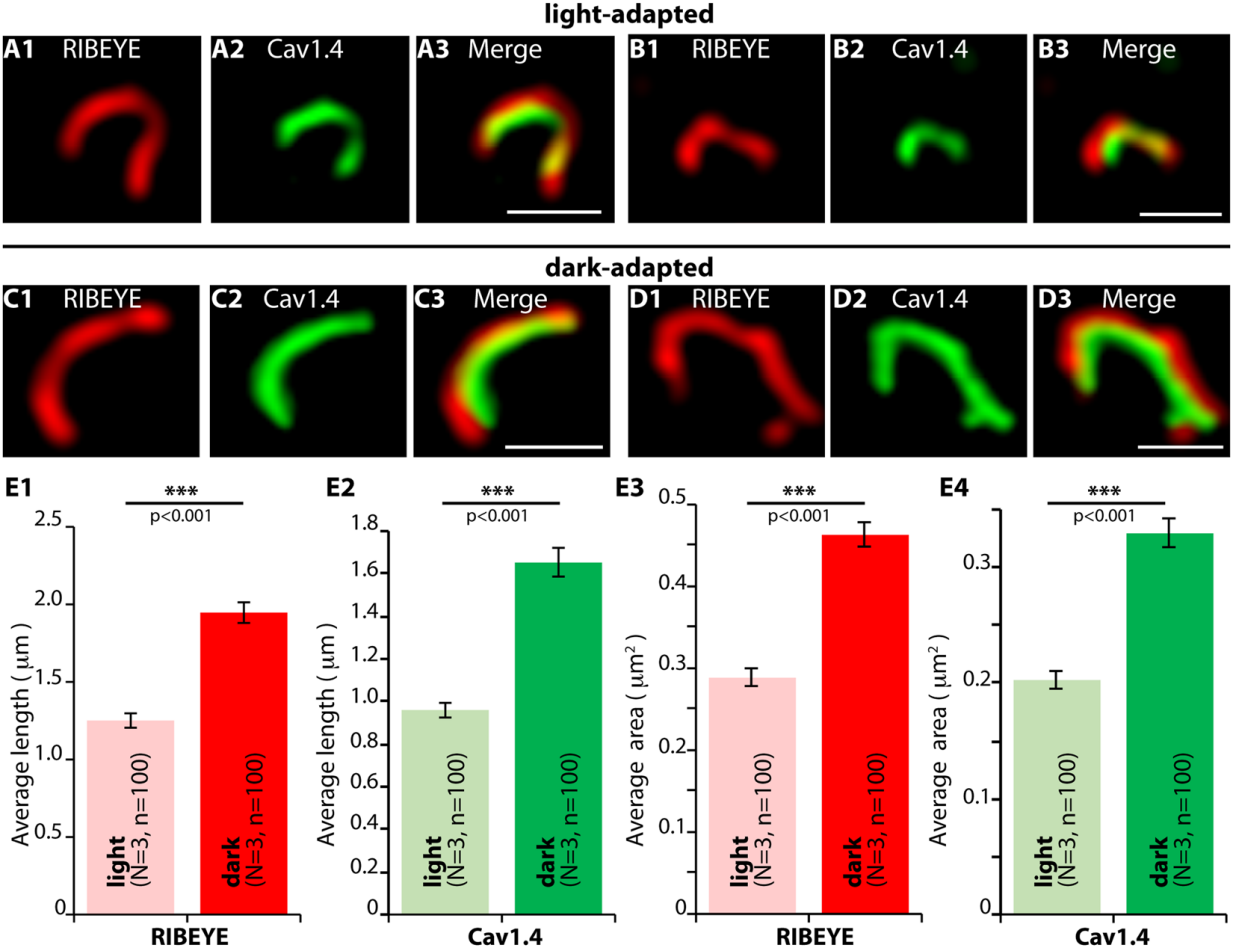
SR-SIM analyses of individual synaptic ribbons from photoreceptor synapses of light- and dark- adapted retinas immunolabelled with polyclonal antibodies against RIBEYE (U2656^14^) and mouse monoclonal antibodies against Cav1.4 (16D9). (**A**–**D**) Representative SR-SIM images from light- and dark-adapted retinas, (**E**) quantitative analyses of the synaptic ribbons/Cav1.4 length and area measurements (mean ± S.E.M.) obtained from super-resolution measurements (**E1**–**E4**). Abbreviations: OPL, outer plexiform layer. Scale bars: 1 μm.

**Figure 6:**
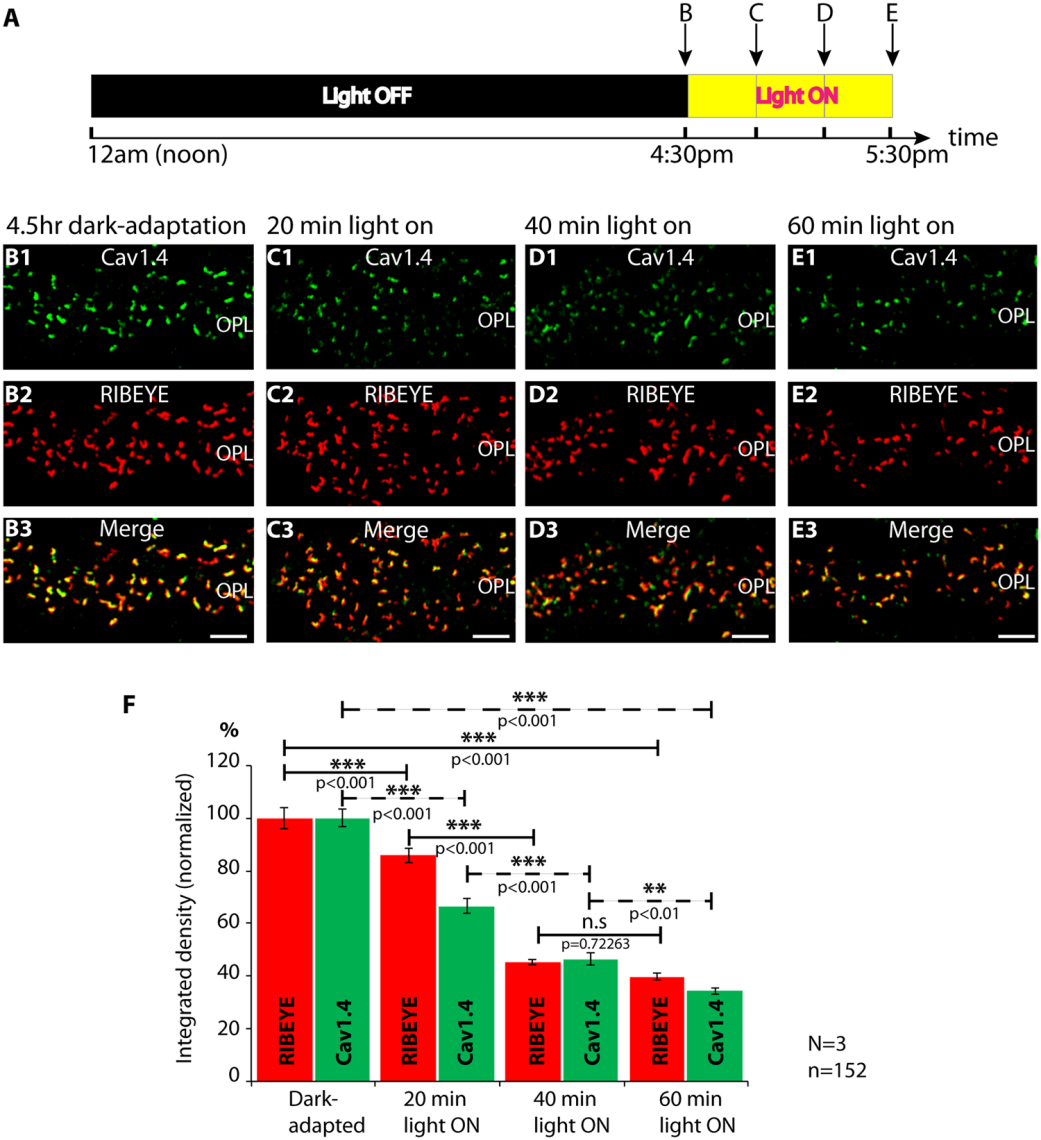
Mice were adapted from 12 am (noon) to 4:30 pm (4.5 hrs) in complete darkness. At 4:30pm in the afternoon, mice were either directly sacrificed in complete darkness or exposed to light for the indicated time periods before sacrificing the mice at ambient light (summarized in **A**). (**B**–**E**) show representative confocal images. In (**F**), immunosignals were quantified (mean ± S.E.M). Within 40 min of light-adaptation, ribbon length returned from dark values with elongated ribbons to light values with shortened ribbons. The increased darkness-induced Cav1.4 length also decreased by illumination. For Cav1.4, returning to light values took slightly longer than for ribbons. The Cav1.4 immunosignals at 40 min post illumination were still slightly higher in comparison to Cav1.4 immunosignals 60 min post illumination. Cav1.4 was visualized with 16D9 mouse monoclonal antibody; RIBEYE with U2656 rabbit polyclonal antibody. Values are mean ± S.E.M. Abbreviations: OPL, outer plexiform layer. Scale bars: 5 μm.

We used super-resolution microscopy (SR-SIM), to further explore the reason for the increased RIBEYE/Cav1.4 immunosignals in photoreceptor synapses of dark-adapted retinas (Fig. 5). For the SR-SIM analyses we focused on ribbons/active zones of rod synapses that can be discriminated from ribbons of cone synapses by the size and shape of the ribbon and by their position in the OPL (see methods). Representative SR-SIM images from light- and dark-adapted rod photoreceptor wildtype synapses are shown in Fig. 5A–D. Quantitative SR-SIM analyses revealed that the synaptic ribbons were longer in dark-adapted rod photoreceptors (Fig. 5E1). Similarly, also the length of the Cav1.4 channel clusters was increased in the dark (Fig. 5E2). For dark-adapted retinas (Fig. 5E1,E2), we observed a mean ribbon length of 1.95 ± 0.07 μm and a mean Cav1.4 length of 1.65 ± 0.07 μm; for light-adapted retinas (Fig. 5E1,E2) a mean ribbon length of 1.25 ± 0.05 μm and a mean Cav1.4 length of 0.96 ± 0.04 μm. Thus, the increase in the length of synaptic ribbons and the associated active zone (Fig. 5A–E) is most likely the main reason for the increased RIBEYE/Cav1.4 immunosignals in dark-adapted photoreceptors (Fig. 4). In support of this hypothesis, the increase of RIBEYE/Cav1.4 immunosignal intensities in dark-adapted rod photoreceptor synapses (Fig. 4C; increase of confocal RIBEYE/Cav1.4 immunosignal intensity by ≈80% in the dark in comparison to the light [light values set to 100%]) increased in the same range as the ribbon/Cav1.4 signal length measured by SR-SIM in dark-adapted rod photoreceptors (Fig. 5E1,E2; increase for ribbon length by ≈ 60% and increase for Cav1.4 signal length by ≈70% in the dark in comparison to the light). Ribbon- and Cav1.4 area sizes in rod photoreceptors were similarly altered (mean ribbon area: 0.29 ± 0.01 μm^2^ (light); 0.46 ± 0.02 μm^2^ (dark); mean Cav1.4 area: 0.2 ± 0.01 μm^2^ (light); 0.33 ± 0.01 μm^2^ (dark); Fig. 5E3,E4). The experiments described in Figs. 3 and 4 pointed to an illumination-dependent regulation of both ribbon length and size of presynaptic Cav1.4 clusters. In order to get insights into the time scale in which these changes of ribbon length and Cav1.4 dynamics occur, animals were dark-adapted for 4.5 hrs and subsequently re-exposed to light, as summarized in Fig. 6A. Then, we measured at which time points after the onset of light we could observe a change of RIBEYE and Cav1.4 immunosignal intensities at the photoreceptor synapses in the OPL (Fig. 6B–E). First illumination-induced decreases of RIBEYE and Cav1.4 immunosignal intensities were already evident 20 min after the onset of light (Fig. 6F). The strongest decrease was observed in the time window between 20 min and 40 min after switching on the light (Fig. 6F). At 60 min after light exposure RIBEYE and Cav1.4 immunosignals displayed the strongest reduction of the respective immunosignal intensities in the analyzed time window (Fig. 6F). These data indicate that the illumination-dependent remodeling of photoreceptor active zone proteins can happen within a timescale of a few minutes.

**Figure 7:**
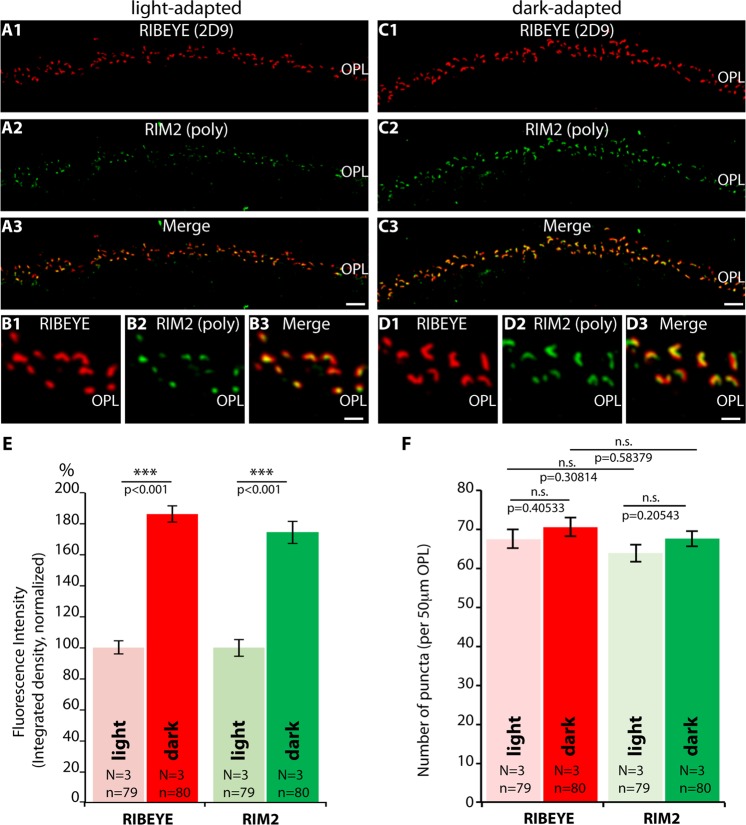
Confocal analyses of photoreceptor synapses from light- and dark-adapted retinas immunolabelled with mouse monoclonal antibodies against RIBEYE (2D9^44^) and rabbit polyclonal antibodies against RIM2. (**A**–**D**) Representative confocal images. (**E**,**F**) quantitative analyses of the strength of the immunosignals (**E**; integrated density; plotted as mean ± S.E.M. In (**E**,**F**)) and number of immunoreactive puncta (**F**). Abbreviations: OPL, outer plexiform layer. Scale bars: 5 μm (**A**–**D**).

**Figure 8:**
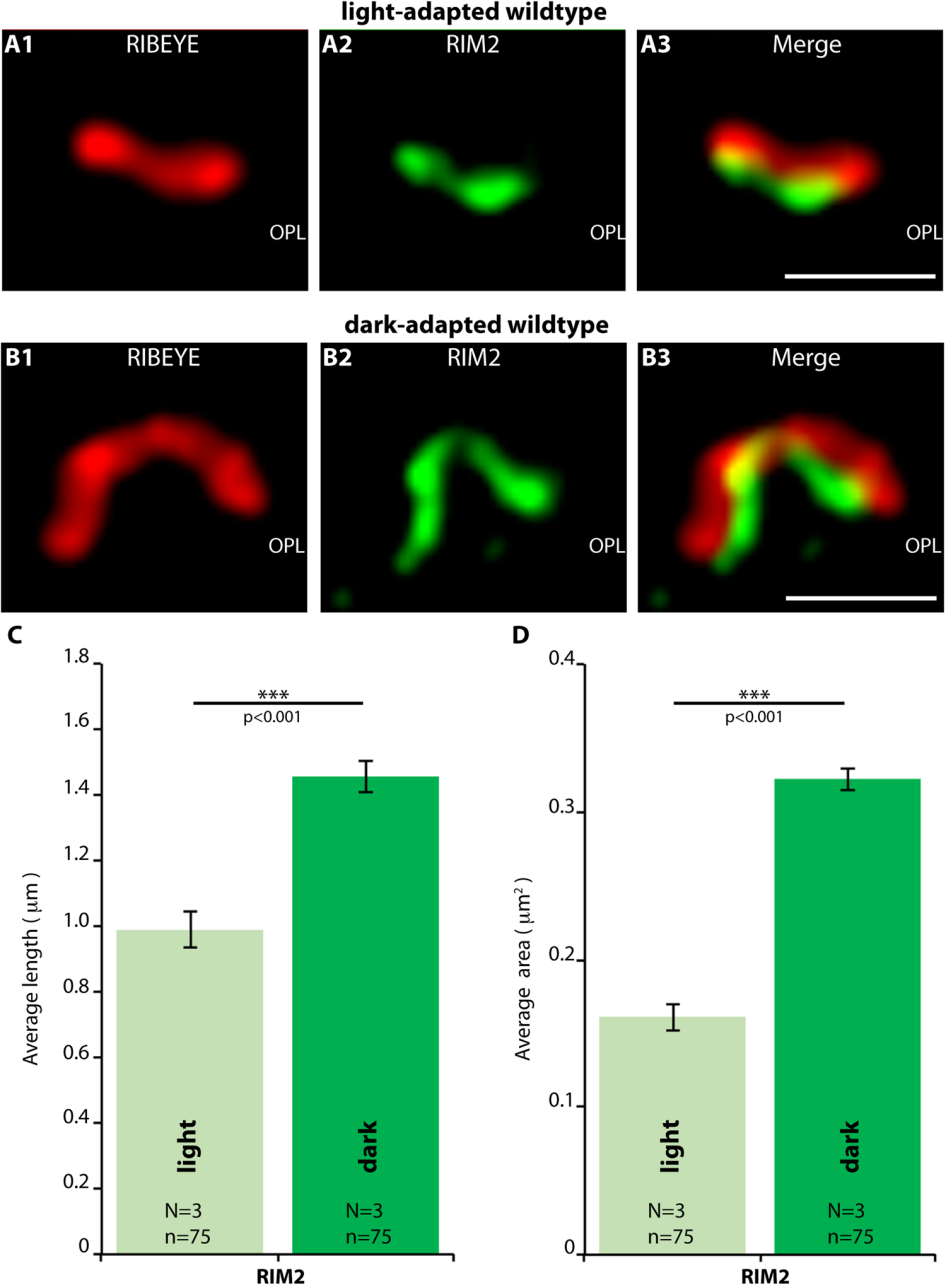
SR-SIM (**A**,**B**) analyses of rod photoreceptor synapses in the OPL of wildtype retinas immunolabelled with rabbit polyclonal antibodies against RIM2 and mouse monoclonal antibodies (2D9) against RIBEYE. The length (**C**) and the area (**D**) of RIM2- active zone protein clusters are quantified. Values are mean ± S.E.M. Abbreviations: OPL, outer plexiform layer. Scale bars: 1 μm (**A**,**B**).

Next, we asked whether RIM2, a major long RIM variant of the active zone of rod photoreceptor synapses, also showed an increased darkness-induced recruitment to the active zone as we observed for Cav1.4. Both proteins are enriched in the active zone and interact directly or indirectly with each other^1,2^. Therefore, we immunolabelled resin sections from light- and dark-adapted retinas with mouse monoclonal antibodies against RIBEYE (2D9) and rabbit polyclonal antibodies against RIM2 and analyzed them by confocal microscopy (Fig. 7A–D). In fact, similar to Cav1.4, the RIM2 immunosignals were also increased in rod photoreceptor synapses of dark-adapted retinas in comparison to light-adapted retinas as judged by confocal microscopy (Fig. 7E). Similarly, as also demonstrated for Cav1.4, the increased RIM2 immunosignals in dark-adapted rod photoreceptors were mainly due to an increased intensity of the RIM2 immunosignals in the OPL (Fig. 7E) but not due to a difference in the number of active zone puncta in the OPL (Fig. 7F). The number of RIM2 puncta in the OPL were not statistically different between light- and dark-adapted samples (Fig. 7F). Thus, both RIM2 as well as Cav1.4 appeared to be more strongly enriched in the active zones of dark-adapted rod photoreceptor synapses in comparison to light-adapted retinas. SR-SIM analyses (Fig. 8) revealed that this increased darkness-induced enrichment of RIM2 observed by confocal microscopy (Fig. 7) resulted from an increase in the length of RIM2 clusters at the active zone. Similar to Cav1.4, the average RIM2 length and RIM2 area was larger in rod photoreceptor synapses of dark-adapted retinas in comparison to light-adapted retinas (Fig. 8C,D). Representative SR-SIM images are shown in Fig. 8A,B.

### The illumination-dependent modulation in the size of RIM2/Cav1.4 at the active zone of photoreceptor synapses depends upon the presence of the synaptic ribbon

Next, we analyzed whether the synaptic ribbon itself might play a role in the darkness-induced increased recruitment of RIM2 and Cav1.4 to the active zone of rod photoreceptor synapses. For this purpose, we analyzed RIM2/Cav1.4 in rod photoreceptor synapses of RIBEYE knockout mice that lack synaptic ribbons^15^ and explored whether these ribbon-deficient knockout mice also display the described illumination-dependent restructuring of the active zone.

**Figure 9:**
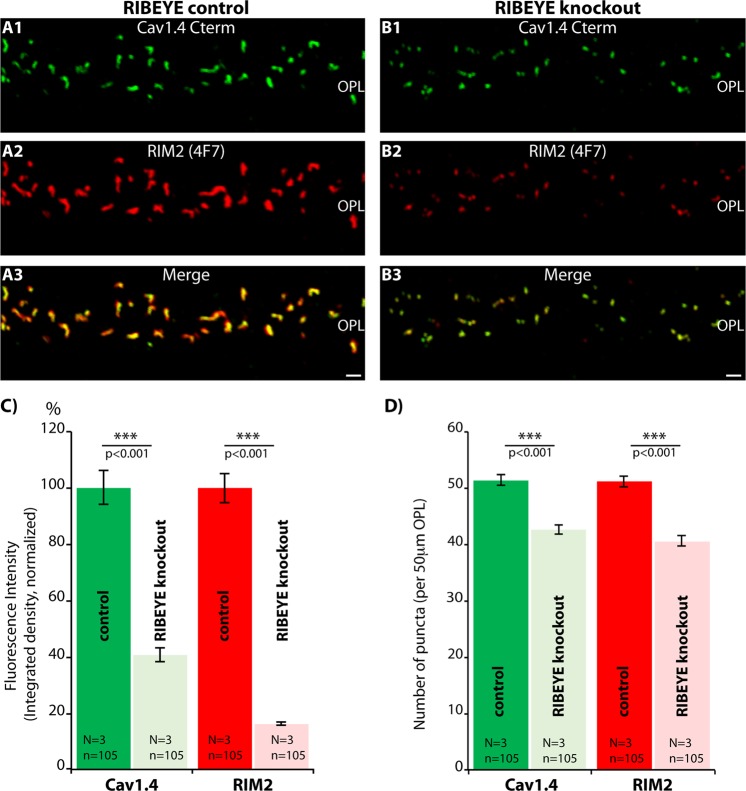
Confocal analyses of photoreceptor synapses from RIBEYE knockout (**B**) and littermate control retinas (**A**) immunolabelled with mouse monoclonal antibodies against RIM2 (4F7) and rabbit polyclonal antibodies against Cav1.4 (Cav1.4 Cterm). (**A**,**B**) Representative confocal images, (**C**,**D**) quantitative analyses of the strength of the respective immunosignals (integrated density in **C**) and number of immunoreactive puncta (in **D**) plotted as mean ± S.E.M. Abbreviations: OPL, outer plexiform layer. Scale bars: 1 μm (**A**–**D**).

We found striking differences for RIM2/Cav1.4 in the active zone of rod photoreceptor synapses of RIBEYE knockout mice in comparison to littermate control mice (Fig. 9). Representative confocal images are shown in Fig. 9A,B. The immunosignal intensities for both Cav1.4 and RIM2 were strongly reduced at the active zone from rod synapses of light-adapted RIBEYE knockout mice (to ≈40% and 20% of the respective immunosignal intensity levels present in light-adapted littermate control mice; Fig. 9C). The number of rod photoreceptor active zones, as judged by Cav1.4 and RIM2 puncta, were also slightly, but significantly, reduced (Fig. 9D).

**Figure 10:**
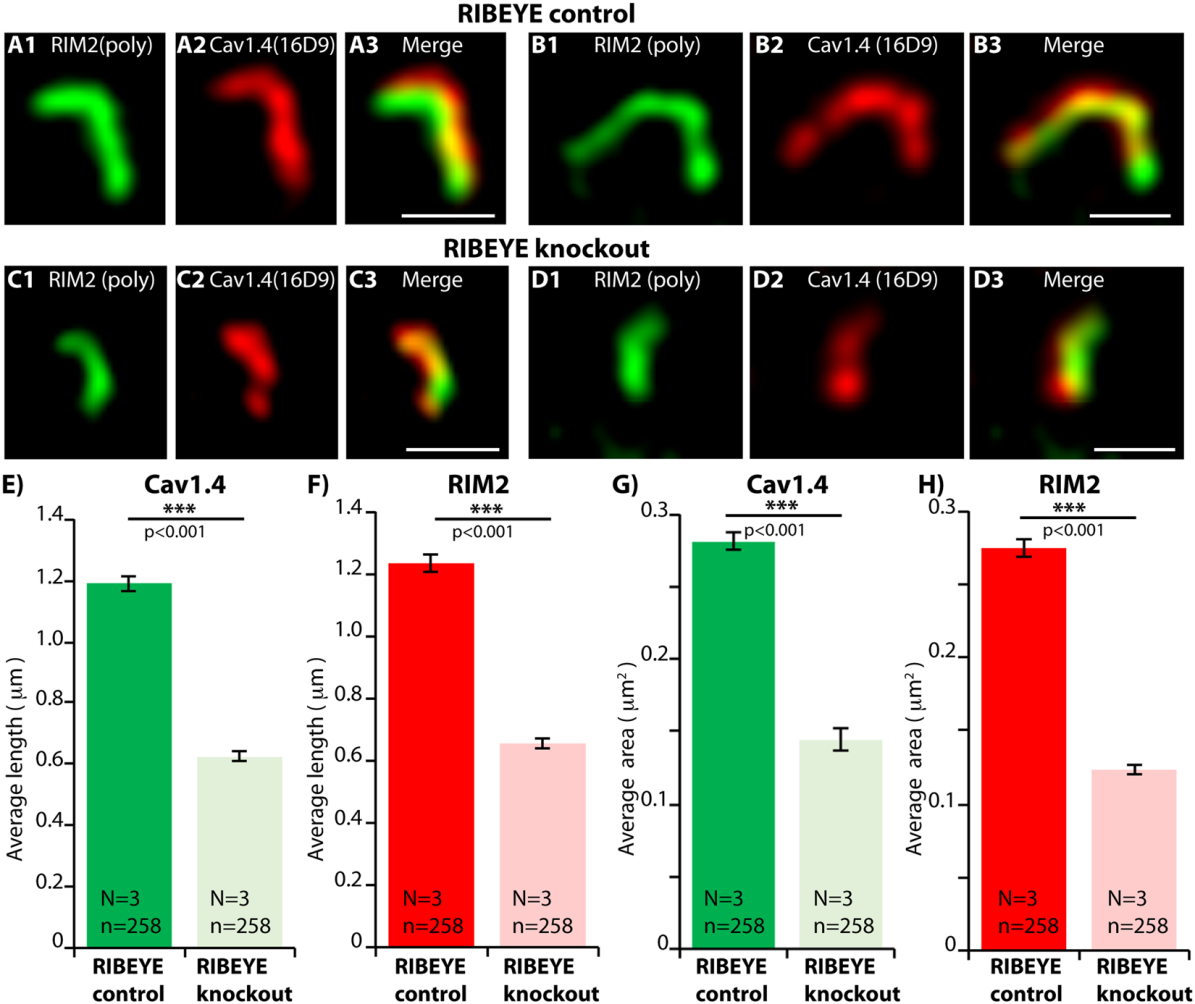
SR-SIM analyses of single synaptic ribbons from rod photoreceptor synapses of RIBEYE knockout and littermate control retinas immunolabelled with rabbit polyclonal antibodies against RIM2 (RIM2poly) and mouse monoclonal antibodies against Cav1.4 (16D9). (**A**–**D**) Representative SR-SIM images, (**E**–**H**) quantitative analyses of the RIM2/Cav1.4 length and area measurements (mean ± S.E.M.) obtained from SR-SIM. Abbreviations: OPL, outer plexiform layer. Scale bars: 1 μm.

The active zones of rod photoreceptor synapses from RIBEYE knockout mice and littermate control mice were further analyzed by SR-SIM. SR-SIM analyses of RIM2- and Cav1.4-immunolabelled active zones of littermate control mice demonstrated that the Cav1.4 and RIM2 immunosignals were perfectly co-aligned along each other and overlapped to a large extend, as expected for proteins that are both located at the active zone (Fig. 10A,B). Also in rod photoreceptor synapses of RIBEYE knockout mice Cav1.4 und RIM2 immunosignals were co-aligned along each other (Fig. 10C,D). But quantitative SR-SIM analyses demonstrated a shorter length of RIM2 and Cav1.4 clusters in rod photoreceptor synapses of RIBEYE knockout mice in comparison to littermate control mice (Fig. 10E–H). Both length as well as area of the respective presynaptic clusters were reduced (mean Cav1.4 length: 1.19 ± 0.03 μm (control); 0.62 ± 0.02 μm (RIBEYE knockout); mean RIM2 length: 1.23 ± 0.03 μm (control); 0.66 ± 0.02 μm (RIBEYE knockout); mean Cav1.4 area: 0.28 ± 0.01 μm^2^ (control); 0.14 ± 0.01 μm^2^ (RIBEYE knockout); mean RIM2 area: 0.27 ± 0.01 μm^2^ (control); 0.12 ± 0.003 μm^2^ (RIBEYE knockout) (Fig. 10E–H).

**Figure 11:**
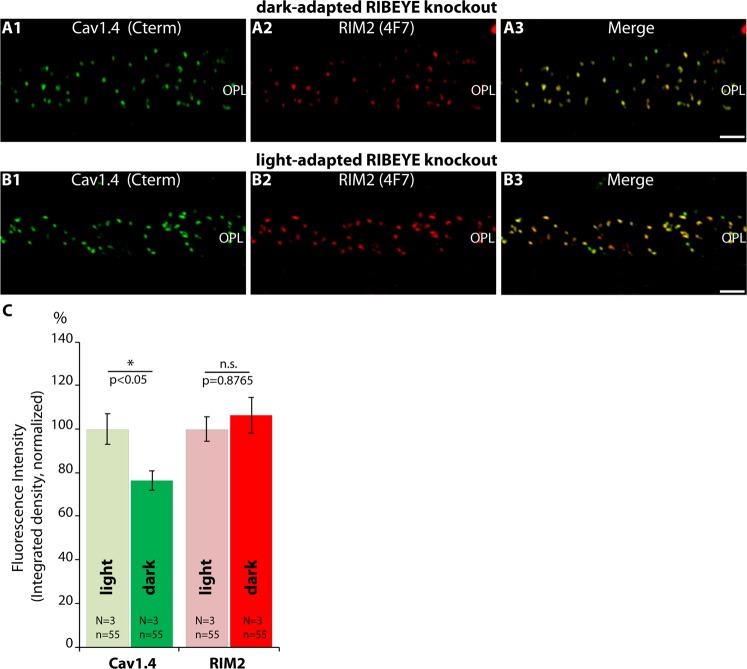
Confocal analyses of photoreceptor synapses from light- and dark-adapted RIBEYE knockout retinas immunolabelled with polyclonal antibodies against Cav1.4 (Cav1.4 Cterm) and mouse monoclonal antibodies against RIM2 (4F7). (**A**,**B**) Representative confocal images, (**C**) quantitative analyses of the strength of the immunosignals (integrated density; plotted as mean ± S.E.M. In (**C**)). Abbreviations: OPL, outer plexiform layer. Scale bars: 5 μm.

**Figure 12:**
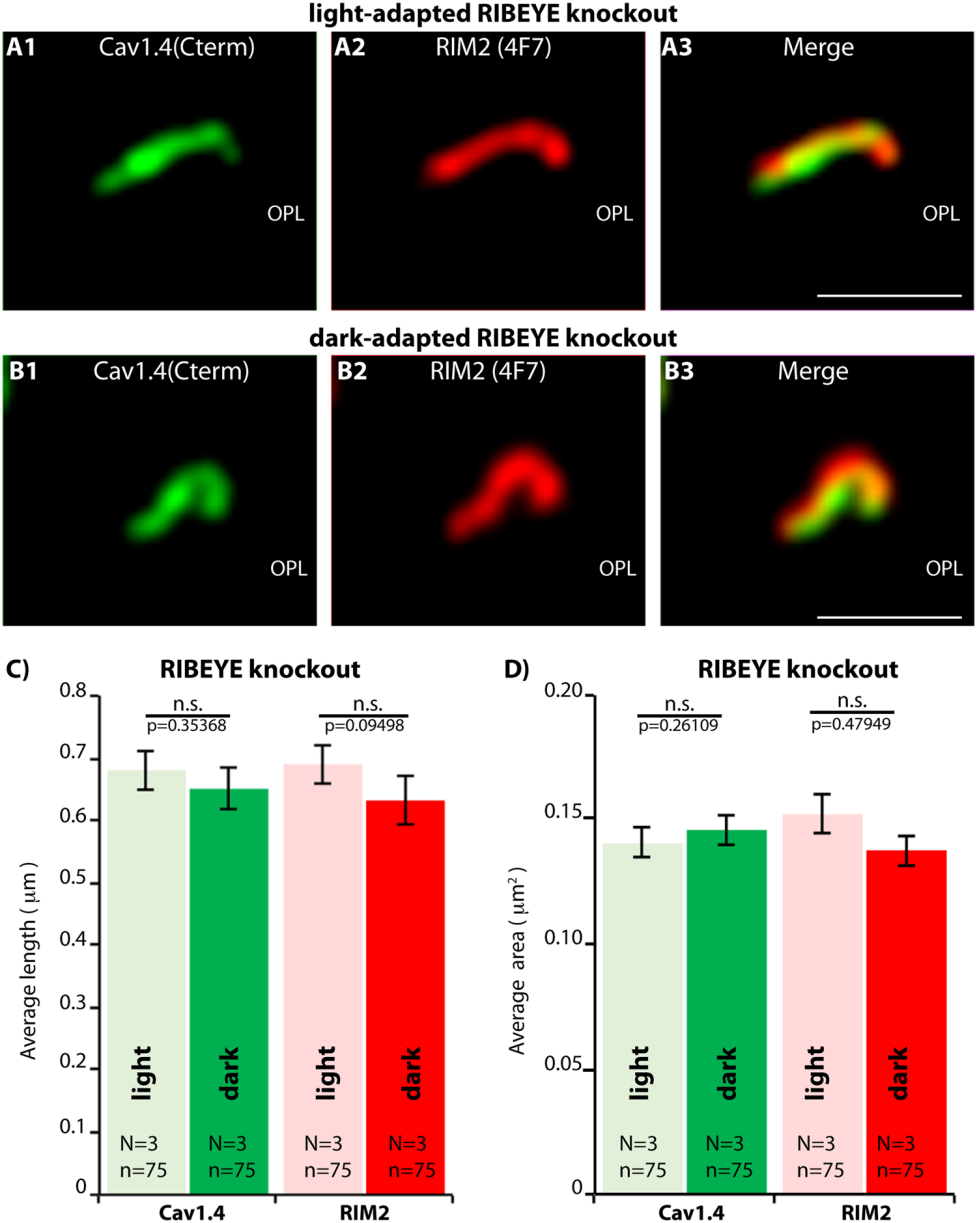
SR-SIM (**A**,**B**) analyses of rod photoreceptor synapses in the OPL of RIBEYE knockout retinas immunolabelled with rabbit polyclonal antibodies against Cav1.4 (Cav1.4 Cterm) and mouse monoclonal antibodies (4F7) against RIM2. The length (**C**) and the area (**D**) of Cav1.4- und RIM2- active zone protein clusters were quantified. Values are mean ± S.E.M. Abbreviations: OPL, outer plexiform layer. Scale bars: 1 μm.

Finally, we compared rod photoreceptor active zones of light- and dark-adapted retinas of RIBEYE knockout mice (Fig. 11). As mentioned above, synaptic ribbons are completely absent in RIBEYE knockout mice^15^. Remarkably, the darkness-induced increase of RIM2/Cav1.4 immunosignals was absent in RIBEYE knockout mice as analyzed by confocal microscopy (Fig. 11). In contrast to C57BL/6J wildtype synapses, Cav1.4 levels even decreased in RIBEYE knockout mice during dark-adaptation (Fig. 11C). Representative confocal images are shown in Fig. 11A,B. The darkness-induced enhanced recruitment of RIM2 to the active zone was also lacking in photoreceptor synapses of RIBEYE mice (Fig. 11C). SR-SIM analyses further revealed that the darkness-induced increase in the size of Cav1.4- and RIM2-clusters at the active zone was also absent in RIBEYE knockout mice in comparison to littermate control mice (Fig. 12). Representative SR-SIM images are presented in Fig. 12A,B; the quantitative SR-SIM analyses are summarized in Fig. 12C,D. These findings suggest a direct impact of synaptic ribbons on illumination-dependent restructuring of the active zone in rod photoreceptor synapses and propose a new role for synaptic ribbon function.

## Discussion

In the present study, we identified illumination-dependent remodeling of the active zone of photoreceptor ribbon synapses in the outer plexiform layer (OPL) of the retina that is dependent upon the presence of the synaptic ribbon. Most of the photoreceptor synapses in the OPL are formed by rod photoreceptors and only a minority of synapses in the OPL (less than 5%) are cone synapses^29,48,49^. Rod synapses have a single large active zone with a single large horseshoe-shaped synaptic ribbon that can be readily identified by immunolabelling against RIBEYE. Due to the large size of the single active zone in rod synapses, these synapses allow high-resolution analyses of single active zone complexes. Using SR-SIM analyses, we showed that synaptic ribbons and active zones (visualized with RIBEYE and Cav1.4 antibodies) are well co-aligned in photoreceptor synapses. Co-labelling of ribbons and active zones (with RIBEYE and Cav1.4 antibodies) and subsequent quantitative analyses suggested a tight coupling between the active zone and the ribbon: Longer ribbons were always associated with larger active zones and, vice versa, smaller ribbons correspondingly with smaller active zones (Fig. 3F2). The ratios between ribbon length and active zone length stayed virtually constant irrespective of the absolute length of the ribbon (Fig. 3F2). Also RIM2, a further component of the photoreceptor active zone, was well co-aligned with RIBEYE (Fig. 8). The proposed interdependence of synaptic ribbons and active zone components, e.g. Cav1.4 and RIM2, is also in line with EM data showing a physical connection between ribbons and active zones^18^ and observations in transgenic zebrafish overexpression models and Cav knockout models^21,22,29,72–76^.

Since synaptic ribbons and active zone appeared to be connected to each other, we asked whether changes in ribbon length also influence the length of the attached active zone, as measured by the length of the active zone Cav1.4 and RIM2 protein clusters. We found that synaptic ribbons (as measured by SR-SIM with anti-RIBEYE) increased in length in rod photoreceptor synapses of dark-adapted retinas in comparison to rod photoreceptor synapses of light-adapted retinas (Fig. 5). Previous EM studies analyzed illumination-dependent changes of the ribbon height in EM cross-sections and found illumination-dependent removal of ribbon material from the distal, non-membrane-associated end of the ribbon^66–68^; but see^69^. These observations are in line with our findings although we focused on the active zone-associated z-dimension of the ribbon (length of the ribbon) and not on the synaptic ribbon height (xy-dimension). We found that the ribbon length was increased in dark-adapted retinas in comparison to light-adapted retinas.

The darkness-induced increase of ribbon length occurred in response to dark-adaptation because both mouse groups (control and experimental groups) were exposed in parallel, at the same time, either to darkness or environmental light. Clearly, circadian signals might play an additional role and also the genetic background of the mouse line could further influence ribbon dynamics. In support of a role of the genetic background, we found slightly different values for Cav1.4 active zone length between our C57BL6/J mice (Fig. 5) and the littermate control mice in the RIBEYE knockout mouse line (Fig. 10). Also, RIBEYE and Cav1.4 length measurements slightly differed when analyzed at different times of the day (Figs. 3 and 5) arguing for a possible influence of circadian signals. The results from Fig. 6 demonstrated that the darkness-induced increase in ribbon length was reversible and can be modulated by light exposure within a few minutes. These data emphasize that synaptic ribbons are not static structures and that the length of the ribbons can be adjusted on a relatively short time scale.

In support of the idea of a tight coupling between the ribbon and the active zone, we found that not only the ribbon increased in length during dark-adaptation but also the active zone. This was shown by co-immunolabelling of the active zone with anti-RIM2 and anti-Cav1.4 antibodies and quantitative SR-SIM measurements.

A previous study^69^ co-stained ribbons and an active zone component of photoreceptor synapses from C57BL/6 mice and did not observe illumination-dependent alterations in ribbon length and active zone length with conventional epifluorescence microscopy (Zeiss Axio Imager.Z1) and apotome imaging. The reason for this discrepancy is unclear but could result from several differences. One reason could be a technical issue. The previous study^69^ used epifluorescence light microscopy and the illumination-dependent changes of ribbon length and active zone length could have escaped the limited resolution of conventional light microscopy. In our study, we applied confocal microscopy and super-resolution microscopy that possess advanced optical resolution. Moreover, in the Fuchs study^69^ light-/dark-stimuli were not applied at the same time of the day for the two experimental groups (light/dark) as in the present study (Figs. 4, 5, 7, 8, 11 and 12), but different time points of the day were compared with each other. This approach could lead to a mix of illumination-dependent and circadian effects that might obscure the illumination-dependent effects. Another reason could be differences in the genetic background of the analyzed mice. Differences in the selected C57BL/6 mouse sub-strain could be relevant for explaining differences in the observed phenotypes. C57BL/6N carry a mutation in the Crumbs CRB1 gene that leads to the rd8 retinal degeneration phenotype^60,77–79^. This degeneration phenotype in C57BL/6N mice strongly affects photoreceptors and might block ribbon- and active zone dynamics. CRB1 gene mutations are also associated with human retinal disorders, including retinitis pigmentosa^80^. Unfortunately, the C57BL/6 sub-strain analyzed in the study by Fuchs *et al*.^69^ was not mentioned in the publication.

In the current study, we used the C57BL/6J sub-strain to avoid the rd8 retinal degeneration phenotype^79^. Furthermore, we also chose C57BL/6J mice because these mice perform better in visual tests than C57BL/6N mice^78^. Interestingly, the C57BL/6J mice carry a loss-of-function mutation in the nicotinamide nucleotide transhydrogenase (Nnt) gene^81^. The Nnt protein catalyses the generation of NADPH from NADH. Differences in NADH levels have been recently proposed to modulate ribbon dynamics possibly by direct binding to RIBEYE^82,83^ that contains an NADH-binding site^14^. Therefore, some differential effects might also be caused by alterations in NADH/NADPH metabolisms in the two different C57BL/6 sub-strains that might modulate ribbon dynamics. Clearly future analyses are required to further investigate these possibilities and to obtain further insights into the underlying molecular mechanisms of ribbon and active zone dynamics.

The darkness-induced, parallel increase of both synaptic ribbon and active zone size raised the possibility that these processes are interdependent and that the ribbons itself could be involved in the darkness-induced increase in active zone size. To directly test this hypothesis, we analyzed the active zone of RIBEYE knockout mice, that specifically lack synaptic ribbons^15^, for illumination-dependent remodeling of the active zone.

We found that the active zones of rod photoreceptors from RIBEYE knockout mice were significantly different from rod synapses of littermate controls (Fig. 9). In the RIBEYE knockout mice, the immunosignals for both RIM2 and Cav1.4 were strongly reduced to about 20% and 40%, respectively, of littermate control levels (Fig. 9C,D). SR-SIM demonstrated that the active zones of rod photoreceptors from RIBEYE knockout mice were significantly smaller (Fig. 10). These data suggest that the ribbon is important to stabilize RIM2 and Cav1.4 at the active zone and support the proposed role of the ribbon in organizing nanodomain coupling of Cav-channels with release-ready vesicles at retinal ribbon synapses^8,15,29,76,84–91^. In support of this hypothesis, afferent inner hair cell ribbon synapses show abnormally organized active zones in RIBEYE knockout mice^92^. Clearly, our current and previous^15^ analyses showed that the active zone is not completely absent in photoreceptor ribbon synapses but only altered. This might explain in part why light-evoked responses of retinal ganglion cells at the systems level are only moderately disturbed^93^.

Remarkably, the ribbon-deficient rod photoreceptor synapses did not show the typical darkness-induced enhanced active zone recruitment of RIM2/Cav1.4 that is present in control mice. These findings suggest that the synaptic ribbon is directly involved in the illumination-dependent recruitment of RIM2/Cav1.4 to the active zones. Interestingly, also in *Drosophila* activity-dependent modulations of the active zone of photoreceptor synapses were observed^94^. The molecular composition of the active zone is crucial for presynaptic signaling^1,95^. The molecular mechanisms of how the ribbon contributes to the darkness-induced increased recruitment of Cav1.4 and RIM2 to the active zone remain to be elucidated. One possible mechanism could be that ribbon-associated vesicles might serve as vehicles for the delivery of active zone proteins. According to this hypothesis, increased exocytosis of these vesicles in the dark, when the photoreceptor is depolarized, could promote formation of larger active zones. Specialized vesicles that transport active zone components have been observed in other systems^96–98^. At the end of the dark period, when light hyperpolarizes the photoreceptors, the active zones become smaller, possibly by endocytotic removal of active zone material. Alternatively, the ribbon might just provide a favorable environment that promotes anchorage of Cav1.4 and RIM2 at the active zone. Larger ribbons would thus stabilize larger Cav1.4/RIM2 clusters and shorter ribbons would favor smaller ones. Clearly, future investigations are needed to characterize the underlying molecular mechanisms.

The increase in ribbon length and active zone length after dark-adaptation likely represents a homeostatic adaptation of the rod photoreceptor synapse. Increased synaptic vesicle exocytosis in the dark^10,16^ will be supported by a larger active zone and an elongated synaptic ribbon. A longer active zone and an elongated synaptic ribbon will provide the active zone with additional release sites and with an increased number of release-ready vesicles.
